# Old and new challenges of HIA Capacity building in Portugal

**DOI:** 10.1093/eurpub/ckac129.102

**Published:** 2022-10-25

**Authors:** L Costa, A Costa, AC Dias Ferreira, P Martín-Olmedo, L Green

**Affiliations:** 1 Health Promotion and Prevention of NCDs, National Institute of Health Dr Ricardo Jorge, Lisbon, Portugal; 2 Instituto de Biossistemas Ciências Integrativas, Faculdade de Ciências, Universidade de Lisboa, Lisbon, Portugal; 3 Public Health, Porto Ocidental Public Health Unit, Porto, Portugal; 4 Cancer Epidemiology, IBs.GRANADA, Granada, Spain; 5 Public Health, Escuela Andaluza de Salud Pública, Granada, Spain; 6 HIA, Public Health Wales, Wrexham, UK

## Abstract

Within the European framework on health, adopted in 2012 by the Member States in the World Health Organization European Region, Health Impact Assessment (HIA) plays a crucial role in achieving the Sustainable Development Goals by supporting decision-makers to address health impacts and inequalities and ensure health gains. In Portugal, the integration of HIA in the Public Health Services (PHS) is still lacking despite of several attempts to implement HIA. In the pandemic and post-pandemic context, Public Health Authorities are busy with a lot of mandatory tasks, and the absence of a national policy framework for HIA that puts into practice the legal obligations arising from the Environment Impact Assessment Directive 2014/52/EU inhibits its sustainable implementation. The National Institute of Health Doutor Ricardo Jorge (INSA) has been involved in capacity building initiatives designed to further support the institutionalization of HIA in Portugal. This presentation outlines the lessons learned from this experience and will identify key facilitators and barriers elements for HIA implementation in specific contexts. The role of PHS in HIA development and execution will be discussed taking into consideration policy dialogue conclusions, and data collected from a survey aim to address the use and perception of this methodology in Public Health Units at the local level. The more recent Portuguese Fundamental Law on Health (Law nº95/2019) provides an important policy support ensuring Health in All Policies (HiAP) and it also opens a new pathway to enable HIA implementation at the national, regional, and local levels. In this respect, the PHS could play a crucial role taking leadership for HIA in Portugal. Novel and old approaches are still withstanding challenges.

